# AI-Aided Search for New HIV-1 Protease Ligands

**DOI:** 10.3390/biom13050858

**Published:** 2023-05-18

**Authors:** Roberto Arrigoni, Luigi Santacroce, Andrea Ballini, Luigi Leonardo Palese

**Affiliations:** 1 Bioenergetics and Molecular Biotechnologies (IBIOM), CNR Institute of Biomembranes, 70125 Bari, Italy; r.arrigoni@ibiom.cnr.it; 2 Interdisciplinary Department of Medicine (DIM), University of Bari Aldo Moro, 70124 Bari, Italy; luigi.santacroce@uniba.it; 3 Department of Clinical and Experimental Medicine, University of Foggia, 71122 Foggia, Italy; 4 Department of Translational Biomedicine and Neurosciences—(DiBraiN), University of Bari Aldo Moro, 70124 Bari, Italy

**Keywords:** HIV-1 protease, HIV protease inhibitors, molecular docking, drug resistance, artificial intelligence, autoencoder

## Abstract

The availability of drugs capable of blocking the replication of microorganisms has been one of the greatest triumphs in the history of medicine, but the emergence of an ever-increasing number of resistant strains poses a serious problem for the treatment of infectious diseases. The search for new potential ligands for proteins involved in the life cycle of pathogens is, therefore, an extremely important research field today. In this work, we have considered the HIV-1 protease, one of the main targets for AIDS therapy. Several drugs are used today in clinical practice whose mechanism of action is based on the inhibition of this enzyme, but after years of use, even these molecules are beginning to be interested by resistance phenomena. We used a simple artificial intelligence system for the initial screening of a data set of potential ligands. These results were validated by docking and molecular dynamics, leading to the identification of a potential new ligand of the enzyme which does not belong to any known class of HIV-1 protease inhibitors. The computational protocol used in this work is simple and does not require large computational power. Furthermore, the availability of a large number of structural information on viral proteins and the presence of numerous experimental data on their ligands, with which it is possible to compare the results obtained with computational methods, make this research field the ideal terrain for the application of these new computational techniques.

## 1. Introduction

In the early 1980s, a new viral infection was recognized, with a pandemic trend that is still ongoing. Acquired immunodeficiency syndrome (AIDS), which has had dramatic clinical implications for many years, is caused by a retrovirus known as human immunodeficiency virus type 1 (HIV-1). It is estimated that this virus has caused around 40 million deaths worldwide, and currently, there are about 38 million patients infected by HIV-1 [1]. Despite significant progress in understanding the immune response to the virus and developing vaccines [2,3,4], the disease is now controlled only by antiviral drugs [5,6]. These molecules affect three viral enzymes: protease, reverse transcriptase, and integrase. The availability of these drugs has radically changed the prognosis and quality of life of HIV-infected patients, so much that the reassuring certainty that HIV-1 infection has become a chronic, but controllable, condition has spread in public opinion. However, this optimistic vision soon collided with the emergence of strains resistant to one or more drugs [7,8,9,10]. The rate of infections with resistant strains has increased significantly in recent years, especially in North America and sub-Saharan Africa, to the point of becoming a real threat to public health.

HIV-1 protease, encoded as part of the Gag-Pol polyprotein in the viral genome, is responsible for the maturation of the Gag-Pol and Gag precursors and has an essential role in the viral replication cycle [11]. For this reason, it was quickly realized that molecules capable of blocking this enzyme were excellent drug candidates for the treatment of AIDS [11,12]. This enzyme is a C2 symmetric homodimer of subunits containing 99 residues [13,14]. Its active site is located at the dimer interface and contains a catalytic aspartate (D25) in the sequence signature DTG. Three regions can be recognized in each monomer: one involved in the enzyme dimerization (residues 1–4 and 95–99); the core region (residues 10–32 and 63–85 of each monomer), which participates to the catalytic site, as well as dimerization; and the flap region consisting of two solvent exposed loops (residues 33–43 of each chain) and two flexible, glycine-rich β-hairpins (residues 44–62). The flexible flaps cap the catalytic triad, and upon substrate- or inhibitor-binding, these plug the active site. The error-prone replication of the HIV-1 rapidly generates a pool of mutant viruses, often resistant to the protease inhibitors [11,15].

HIV-1 protease, which is one of the most important targets of the highly topical research field known as computational virology [16], has been the subject of numerous molecular dynamics studies [17,18,19,20,21,22,23,24,25] and analysis of large crystallographic data sets [26,27,28,29]. These studies suggest that the most stable form of the enzyme is the semi-closed (or semi-open) one, followed by a more tightly closed form, whereas the enzyme in the open flap conformation is difficult to observe.

The aim of this work was to identify new ligands for the HIV-1 protease. We used a strategy based on a first phase of screening assisted by artificial intelligence (AI). The approach used in this phase was essentially the one described in [30], in which two neural networks work in concert. The first is a variational autoencoder (VAE), whose function is to generate a numerical representation of the molecules which can then be used by the second neural network. The latter is trained to associate the VAE numerical representation with a measure of the efficacy of known ligands of our target protein. Once trained, this system can be used for the rapid screening of large numbers of molecules. Subsequently, molecules identified as potential ligands were subjected to molecular docking, and the most interesting ones were validated by molecular dynamics. The advantage of this approach is that it is possible to eliminate many molecules that most likely have no chance of being interesting ligands of the target protein before proceeding with the molecular docking, thus reducing the computational load. Our most promising candidate belongs to a chemical class which, to the best of our knowledge, has not been considered for HIV-1 protease inhibition.

## 2. Materials and Methods

Molecules with known activity on the HIV-1 protease were obtained from ChEMBL [31,32,33] (query target CHEMBL243). The data set was analyzed with *pandas* [34,35] for the presence of duplicates, which were eliminated, and in this case, the one with the best inhibition value was kept.

The neural network (NN) used in this work consisted of two parts: a VAE and a deep feed-forward network (DNN), as described in [30]. NNs were implemented in Keras [36] with TensorFlow [37] as the backend. The VAE consists of two parts, namely the encoder and the decoder. The first one encodes a particular representation of the molecules (in our case, the SMILES representation [38]) in a numerical vector; the second one decodes the same vector in the starting representation. We used VAE because it has the advantage of minimizing the non-coding areas of the latent space (the latter is jargon to indicate the space containing vectors encoded by the VAE). Several VAE implementations are available [39,40,41,42,43], even already-trained ones, which can be used on SMILES strings; in this work, training was carried out as suggested in [42]. Vectors obtained by VAE staring from SMILES representations were used as input for various DNN architectures, which were trained to calculate the pChEMBL values associated with the corresponding molecules. We tried different DNNs, containing from three to nine fully connected layers (dense layers), with or without dropout layers (up to six). The Adam algorithm was used for the optimizer, with mean absolute error as loss function, and the learning rate was controlled with the Keras ReduceLROnPlateau function by monitoring the loss function. Interested readers can find in the Supplementary Materials the pseudo-code to reproduce the DNN used to obtain the results described below. Once trained, the VAE-DNN system was used to predict the binding value (as pChEMBL score) on a data set containing 250,000 molecules (which we will refer to as ZINC250K), coded as non-isomeric SMILES, obtained from ZINC20 [44]; see also [43]. Molecules in ZINC250K with the best and worst predicted pChEMBL scores were selected; each of these two sets initially contained 933 items, and those that generated valid *pdbqt* files in Open Babel [45] were then used for further analysis.

Molecular docking was performed by means of AutoDock Vina 1.2.3 [46,47], essentially as described [48,49]. The PDB [50,51] structure 5IVQ [52] was used as receptor, and its *pdbqt* file was obtained by the AutoDockTools suite [53], with which the hydrogen atoms and Gasteiger-Marsili charges were added [54]. The docking box of dimensions (12.0 Å, 16.0 Å, 16.0 Å) was centered at the coordinates (18.6 Å, 18.6 Å, 6.6 Å).

Molecular dynamics has been performed in NAMD [55] in a water box with 15 Å padding, essentially as described [49,56,57] with few modifications. Briefly, a CHARMM36m force field [58] was used, and parameterization of the protein-ligand complex was carried out by means of CHARMM-GUI using Antechamber for ligand modeling [59,60,61,62,63,64]. Ionic strength and electroneutrality were obtained by adding potassium and chloride ions at a concentration of 150 mM. Periodic boundary conditions and the particle-mesh Ewald (PME) method have been used; the time step was 2 fs. Systems underwent 10,000 conjugate gradient minimization steps followed by 125,000 equilibration steps in canonical ensemble conditions, with the protein–ligand complex fixed, after which 10 ns production runs began in the NpT ensemble (Langevin dynamics at 303.15 K and Nosé-Hoover Langevin piston at 1.01325 bar). Structural analysis was conducted essentially as described in a VMD (version 1.9.3) environment [65,66,67,68].

Numerical calculations were performed using Numpy [69] and Scipy [70] in a Jupyter environment [71]. Graphs were obtained in Matplotlib [72]. The manipulation of SMILES strings, such as the conversion between isomeric and the non-isomeric forms, has been performed using the RDKit software suite [73].

## 3. Results

The main aim of this work was to try to identify new types of molecules capable of binding the HIV-1 protease. Virtual screening can be used in the early stages of the search for new protein ligands to speed up (even enormously) the evaluation of potential candidates. Currently, in the initial stages of in silico screening, molecular docking [74] is the first choice. Although docking is much faster than techniques that start from first principles such as molecular dynamics, being challenged with a large number of ligands requires extremely powerful computing infrastructures, and docking-dedicated databases today contain millions or even billions of entries [32,33,44,75,76,77].

In reality, most of the docking computing time is wasted by the algorithm to evaluate molecules of little chance to be ligands of the target of interest. Therefore, the computational strategy used in this work was based on the initial deployment of an NN for the rapid screening of potentially interesting molecules, consisting of a VAE and a DNN. At the VAE [30,78], previously trained with a sufficiently large database, molecules were presented as SMILES strings and then transformed by this into a numerical (i.e., vectorial) representation. In principle, any kind of molecular representation can be used for the autoencoder training, but representation as SMILES strings, despite being very simple, can obtain good results with a modest computational cost [39,40,41,42,43]. SMILES representation of molecules, whose experimental affinity for the HIV-1 protease is known, were obtained from ChEMBL. Only ligands with reported binding efficiency index (BEI), surface efficiency index (SEI), and pChEMBL value [79,80] were considered, and 5863 items in ChEMBL matched these criteria. After deleting duplicates and entries that cannot be processed by the VAE (exclusion criterion was the length of the SMILES string of the ligand or the impossibility to obtain a latent vector),the final data set was composed of 4299 entries, each consisting of the VAE-generated vectorial representation of the molecule and the associated pChEMBL value. This data set was then split into training set and test set in a ratio of 0.8 to 0.2.

**Figure 1:**
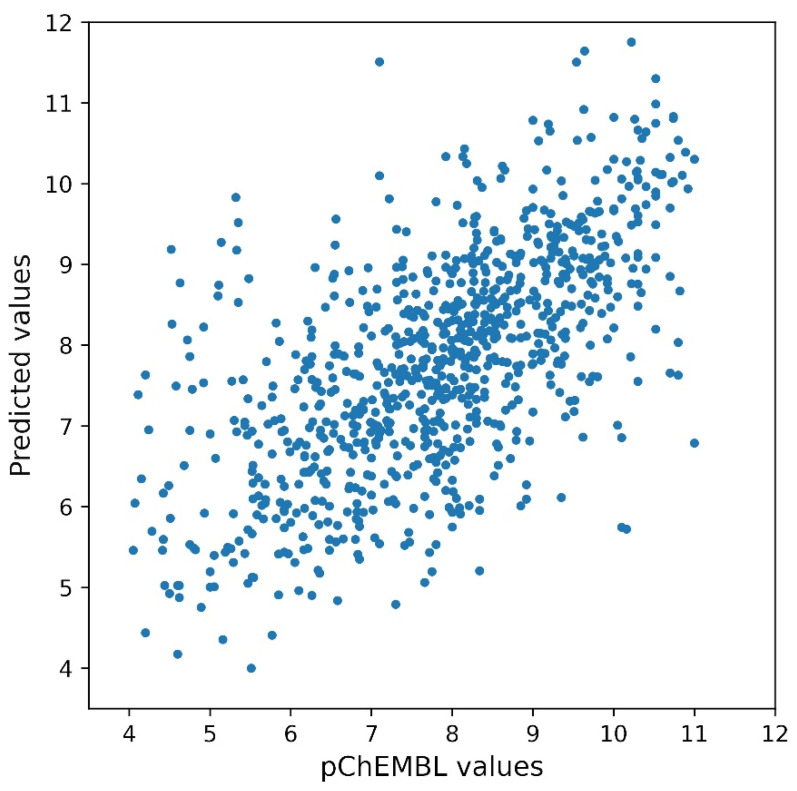
Results of fitting by the DNN described in the text on the ChEMBL test data set. The figure shows the prediction of the neural network described in the text on the ChEMBL test set (see also, Supplementay Data). Experimental data are reported on the horizontal axis, whereas the DNN predicted values are on the vertical one. The best fitting performed in Scipy gives as a result a straight line (not shown) with parameters: slope 0.58698, intercept 3.20243, R-value 0.64671, *p*-value 5^−103^, standard error 0.02363, intercept standard error 0.18865.

The first set was then used to train the DNN to transform the vector representation of molecules into pChEMBL values. Below, we will refer to what was obtained using an eight-layer DNN (see Section 2). This DNN is able to fit the train set almost perfectly: a linear relationship was obtained between the experimental and predicted pChEMBL values (slope 1.0000047, intercept—4.46^−5^, R-value = 0.99999), which is not surprising, since the DNNs are capable of approximating any function [81], however complicated it may be. Obviously, the results on the test set are not so impressive, but a linear relationship between the experimental values and those estimated by the DNN is clearly visible, as reported in Figure 1. The linear fitting led to the following results: slope 0.58698, intercept 3.20243, R-value 0.64671, *p*-value 5^−103^, standard error 0.02363, intercept standard error 0.18865.

No tendency to over-fitting (which could lead to a loss of ability to generalize by the DNN) was observed with any of the architectures used (not shown). The trained DNN was then used to predict the pChEMBL values of a larger data set. Here, we used the ZINC250K data set containing 250,000 molecules. The obtained pChEMBL scores range from a minimum of 3.02 to a maximum of 13.49. Interested readers can find the composition of this dataset in the Supplementay Data, where SMILES codes used for the calculation of the vector representation and the corresponding calculated score are reported.

The good performance of DNN on the test set, as reported above, does not necessarily imply that it is able to obtain similar results on random sets of molecules, such as the ZINC250K data set. This is because databases reporting experimental screening results for a particular target are most likely affected by bias. In general, an excess of particular classes of molecules, or functional groups, is expected in these data sets: if a class of molecule is known to be a good ligand for a particular target protein, many other molecules of the same class, or containing the same functional groups, will have been tested experimentally in an attempt to find new and better ligands, or in an attempt to find relationships between ligand structures and their activity (QSAR studies). However, even when there should not be this kind of bias, the chemical space is so enormous that a data set of a few thousand molecules is focused on only a small fraction of the possible ones (therefore, the data set is biased, inevitably; see Section 4).

**Figure 2:**
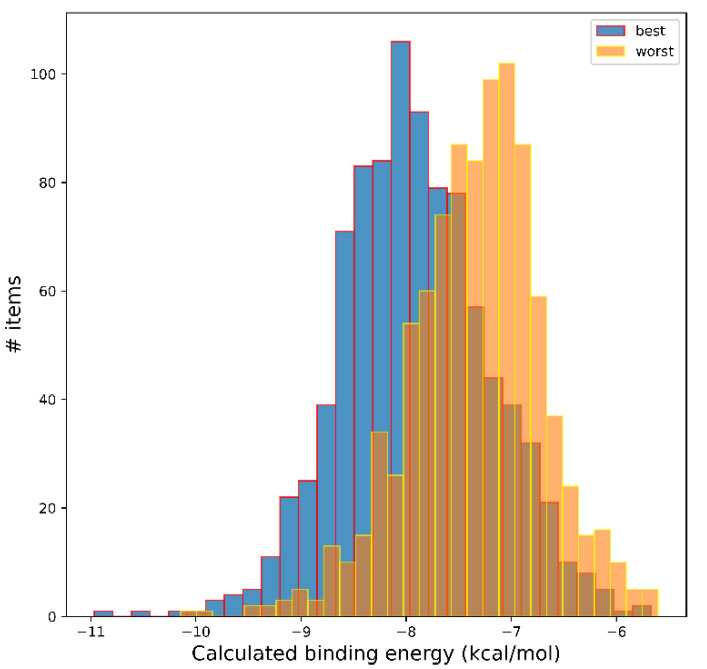
Molecular docking analysis of the best and worst predicted ligands by the DNN in the ZINC250K data set. The histogram shows the number of entries in the binned data of the two data sets, according to the calculated binding energies in the docking experiments. AutoDock Vina software suite was used for docking, as detailed in the main text. Numerical analysis was performed in Numpy.

Keeping what has been reported above in mind, we evaluated the validity our AI system predictions by considering how the predicted best (and worst) ligands performed in a completely different computational setup, namely molecular docking. We selected as best predicted ligands those with calculated pChEMBL values ≥ 10.0, and as worst predicted ligands, an equal number of molecules with the lowest predicted pChEMBL value (each of the two data sets containing 933 elements). These molecules were subjected to molecular docking on an HIV-1 protease template by means of AutoDock Vina, as detailed in Materials and Methods. The results of this analysis are shown in Figure 2, which reports the distribution of the calculated binding energies (BEs).

Even if there is a certain overlap, the two distributions are clearly different, both as average and as extreme values. Molecules predicted as best ligands by the DNN are, on average, better than those predicted as bad ligands: the average BE of the best ligand subset is 7.92 kcal/mol (standard deviation 0.71), whereas for the worst subset, it is 7.33 kcal/mol (standard deviation 0.64). The Kolmogorov–Smirnov test, performed assuming as the null hypothesis that the two distributions are identical, returns as distance 0.38645 and *p*-value 2.15438^−62^. This may be taken as evidence against the null hypothesis and, consequently, that the two distributions are not identical. This suggests that the DNN we trained can be used effectively to select ligands which are more likely to be good ligands, avoiding wasting computational time on molecules that probably will not give any interesting outcome. This result is remarkable considering the diversity of the training data set obtained from ChEMBL and the ZINC250K data set.

To further validate the results obtained, we analyzed a series of molecules using molecular dynamics. We focused our attention on molecules that were part of the best set based on what was predicted by the DNN, and with a BE after molecular docking better than that of the ligand present in the 5IVQ structure. This ligand is a potent inhibitor, and probably a good candidate for clinical development; its chemical name is methyl N-[(2S)-1-[3-[(2R)-morpholin-2-yl]propylamino]-1-oxo-3,3-diphenylpropan-2-yl]carbamate [52]. Molecular docking of this molecule on its own receptor, carried out using the protocol reported in Section 2, leads to a calculated BE of 9.149 kcal/mol. Besides the BE value, we also manually evaluated the goodness of the docking obtained; it should be remembered that we used the procedure in which the receptor is rigid, so we carefully considered the presence of clashes and how extensively the ligand molecule occupied the region of the active site. By these criteria, we considered for molecular dynamics the following ZINC250K molecules: ZINC1040457718 (BE 10.965 kal/mol), ZINC948788229 (BE 10.534 kcal/mol), ZINC991374169 (BE 10.156 kcal/mol), ZINC987999904 (BE 9.642 kcal/mol). Moreover, we queried both ZINC20 and PubChem for molecules similar to ZINC1040457718 (the best ligand after molecular docking) using the built-in Tanimoto similarity search engines. We obtained 124 molecules which were then subjected to molecular docking on the HIV-1 protease (not shown). Many of these molecules have shown good affinity for the enzyme, but one showed a very interesting affinity (ZINC31942116, BE 12,231 kcal/mol), so we also considered this molecule for molecular dynamics. As a control, the dynamics of the holoenzyme and of the enzyme bound to its crystallographic ligand after docking were also performed.

**Figure 3:**
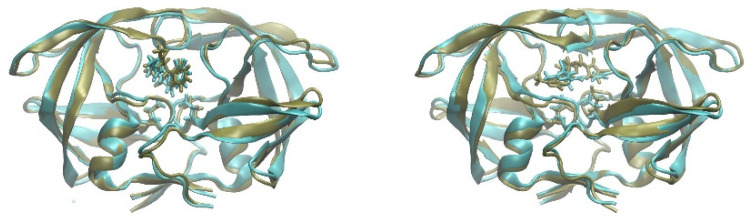
Structures of the protease–ligand complexes. The starting simulation structures of the protease–ligand complexes are reported in cyan, whereas those after 10 ns simulation are reported in tangerine. Starting simulation structures were obtained by minimization of the protease–ligand complex after molecular docking, as described in Section 2. The D25 residue of the HIV-1 protease is highlighted as licorice in all structures. Left structures refer to the HIV-1 protease bound to the crystallographic ligand reported in 5IVQ (see text); right structures refer to the HIV-1 protease bound to ZINC991374169.

After 10 ns of simulation, all the molecules considered were still in the active site of the enzyme. However, taking these data as significant evidence of the fact that we are dealing with a good ligand candidate is absolutely not sufficient. In our case, inspection of the molecular dynamics trajectories suggested a simple criterion to evaluate the effectiveness of the protein-ligand interaction: all the protein-ligand complexes considered, except one, showed a higher root mean squared deviation (RMSD) of the protein atoms than that obtained with the crystallographic ligand. Indeed, whereas in the case of the protease bound to its crystallographic ligand, an RMSD equal to 1.24 ± 0.46 Å was observed (the holoenzyme showed a similar RMSD to that of the crystallographic ligand, 1.23 ± 0.47 Å), in the case of the ligands listed above, the value was higher (for example, 1.40 ± 0.55 Å for ZINC987999904, 1.58 ± 0.69 Å for ZINC31942116), except for ZINC991374169, for which an RMSD of 1.28 ± 0.48 Å was obtained. However, even more interesting was the aspect of the protease, which remained in the closed conformation of the active site in the case of the crystallographic ligand and of ZINC991374169 (see Figure 3), whilst it assumed a swollen, semi-open conformation with all the other ligands (not shown), thus justifying the slightly higher value of the RMSD without a loss of overall stability of the protein. Furthermore, whereas the crystallographic ligand and ZINC991374169 after 10 ns were very close to the starting position (obtained by docking), as reported in Figure 3, in the case of the other molecules, the final position was very different, with a considerable mobility of these molecules into the semi-open active site during the simulation (not shown). All this suggests that only ZINC991374169 could be considered a plausible candidate for experimental validation. The structures of ZINC991374169 and of the crystallographic ligand are shown in Figure 3.

**Figure 4:**
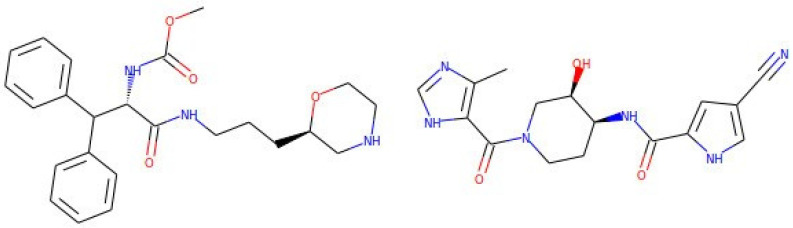
Structural formulas of molecules with best result after molecular dynamics. The structure of the crystallographic ligand present in the PDB entry 5IVQ is shown on the right; this molecule is reported in ZINC20 as ZINC584904731. On the left, the structure of the ligand identified in this work as best candidate, ZINC991374169.

The results of the first phase of screening were validated by means of classical techniques of computational biochemistry, i.e., docking and molecular dynamics (Figure 4).

## 4. Discussion

The search for new molecules capable of blocking the replication of pathogens, including viruses, has become a pressing clinical concern [8,9], and future projections indicate that if the current trend is not reversed, infectious diseases will once again be one of the main, if not the main, causes of death. Our interest has turned to one of the main drug targets for AIDS therapy, namely the HIV-1 protease. Several drugs are used today in clinical practice whose mechanism of action is based on the inhibition of this enzyme [11,13]. However, after years of use, even these molecules are beginning to be interested by resistance phenomena, with the serious possibility of a dramatic throwback to the early years of the AIDS pandemic. Very often, the search for new molecules capable of binding specific target proteins starts with computational methods that allow for the rapid screening of a large number of potential candidates. The vastness of the chemical space, or rather, the more than astronomical number of possible molecules, make the research always and only partial [82,83,84], hence the need for techniques capable of analyzing a large number of molecules quickly and with the least possible computing power. The new AI techniques that are increasingly gaining ground have the potential to greatly accelerate virtual screening processes [78].

We used a simple AI system for the initial screening of a data set containing a number of candidate molecules. Using this system, it has been possible to identify, in a short time, a new molecule with remarkable affinity in silico for the active site of the HIV-1 protease. As expected, this AI system can be used only for a first screening, but it should be noted, however, that this is a general problem: if we were really able to predict with absolute certainty whether a molecule is an effective drug, most of the problems of medicine would already be solved. However, it should be noted that computational analysis of protein-ligand interactions is an old and still not completely solved problem [85], as well as the evaluation of binding affinities and the effects on protein dynamics and functions. Bearing in mind the above limitations, the results we obtained for ZINC991374169 are interesting, particularly when compared with that of the inhibitor bound to the HIV-1 protease used as a template for molecular docking (the PDB entry 5IVQ). Our results suggest that this molecule could be an interesting scaffold, which deserves to be explored.

Besides the particular molecule identified here, whose real efficacy as inhibitor of the HIV-1 protease will be established only experimentally, this work shows how it is possible to use AI-based computational techniques on protein targets to significantly reduce the search space in virtual screening. It is also interesting to underline the criterion (self-contained in a computational context) for the final acceptance of a good candidate ligand, i.e., the evaluation of the RMSD of the protein target compared to an adequate control. We wish to underline once again how this evaluation/validation criterion of potential ligands can be used when the structural (mainly crystallographic) data of control ligands whose activity is known are available. The availability of numerous viral protein structures, combined with the large number of experimental inhibition data, make the search for new antivirals an extremely interesting field of application for these computational techniques.

## Data Availability

All relevant data obtained in this work and software not freely available online are reported in the text and in the Supplementary Materials. Any other data can be provided by the corresponding author upon reasonable request.

## Supplementary Materials

The following supporting information can be downloaded at: https://www.mdpi.com/article/10.3390/biom13050858/s1, Figure S1: ChEMBL test data set and SMILES codes used for the calculation of the vector.

## Abbreviations

The following abbreviations are used in this manuscript:

NN	Neural network
DNN	Deep feed-forward network
VAE	Variational autoencoder
QSAR	Quantitative structure–activity relationship
RMSD	Root mean squared deviation
