# Supplementary material for: AI-Aided Search for New HIV-1 Protease Ligands
Source: Biomolecules. 2023 May 18;13(5):858. doi: 10.3390/biom13050858 (PMC10216636; doi:10.3390/biom13050858)
Supplement: Supplementary file 1 [file biomolecules-13-00858-s001.zip › DNN.pdf]

```
import tensorflow as tf
from tensorflow import keras

from tensorflow.keras.models import Model
from tensorflow.keras.layers import Input
from tensorflow.keras.layers import Dropout
from tensorflow.keras.layers import Dense
from tensorflow.keras import regularizers

from keras.models import Sequential

from sklearn.model_selection import train_test_split



CheMBL_HIV_model2 = Sequential()
CheMBL_HIV_model2.add(Dense(latent_dim, input_shape=(latent_dim,), activation="relu"))
CheMBL_HIV_model2.add(Dense(latent_dim, activation="relu"))
CheMBL_HIV_model2.add(Dense(latent_dim, activation="relu"))
CheMBL_HIV_model2.add(Dense(latent_dim, activation="relu"))
CheMBL_HIV_model2.add(Dense(latent_dim, activation="relu"))
CheMBL_HIV_model2.add(Dense(latent_dim, activation="relu"))
CheMBL_HIV_model2.add(Dense(latent_dim, activation="relu"))
CheMBL_HIV_model2.add(Dense(1))
CheMBL_HIV_model2.compile(optimizer="adam", loss="mse", metrics=["mae"])


from tensorflow.keras.callbacks import History, ReduceLROnPlateau
h = History()

X_train, X_test, y_train, y_test = train_test_split(X_latent, y_latent,
test_size=0.2,random_state=42)
# X_latent and y_latent are the encoder generated vectors and the experimental binding values

rlr2 = ReduceLROnPlateau(monitor='loss', factor=0.5, patience=10, min_lr=0.000001, verbose=1,
min_delta=1e-5)

CheMBL_HIV_model2.fit(X_train, y_train, batch_size=128, epochs=10000, callbacks = [rlr2])
```

Figure S1: ChEMBL test data set and SMILES codes used for the calculation of the vector.
